# A New Challenge of Antibiotic-Resistant Bacteria: Carbapenem-Resistant *Enterobacter cloacae* Complex in a One Health Perspective

**DOI:** 10.3390/microorganisms14030594

**Published:** 2026-03-06

**Authors:** Huina Wang, Jingyi Han, Yuhui Li, Dong Ding, Xuewen Li

**Affiliations:** 1 Department of Environment and Health, School of Public Health, Cheeloo College of Medicine, Shandong University, Jinan 250012, China; whn230516@163.com (H.W.); 15263953117@163.com (Y.L.); 2 Department of Thoracic Surgery, Qilu Hospital of Shandong University, Jinan 250012, China; 202462000588@email.sdu.edu.cn; 3 College of Public Health, Zhengzhou University, Zhengzhou 450001, China; ding251718@163.com

**Keywords:** *Enterobacter cloacae* Complex, carbapenemase, antibiotic resistance, One Health, transmission

## Abstract

Carbapenem-resistant *Enterobacter cloacae* Complex (CRECC) has emerged as an important multidrug-resistant pathogen in healthcare settings, although it has historically received less attention than carbapenem-resistant *Klebsiella pneumoniae* and other major carbapenem-resistant *Enterobacterales* (CRE). Recent epidemiological reports from several regions indicate increasing detection rates of CRECC in tertiary hospitals, where it is associated with bloodstream infections, pneumonia, urinary tract infections, and prolonged hospitalization. The dissemination of carbapenemase genes, particularly *bla_NDM_*, *bla_KPC_*, and *bla_OXA-48-like_*, carried predominantly on conjugative plasmids (e.g., IncFII, IncX3, IncL), represents the primary resistance mechanism, often accompanied by porin loss and efflux pump overexpression. High-risk clones such as ST171 and ST78 contribute to nosocomial persistence and outbreak potential. Beyond clinical settings, CRECC and related resistance determinants have been reported in companion animals, livestock, food products, wastewater systems, and natural aquatic environments. Although most available studies examine these sectors separately, the recurring detection of genetically related resistance genes and plasmid types suggests potential epidemiological links that warrant integrated surveillance. Environmental reservoirs, particularly hospital effluents and wastewater treatment systems, may facilitate the maintenance and dissemination of resistance genes. This review synthesizes current evidence on the epidemiology, resistance mechanisms, and evolutionary dynamics of CRECC in human, animal, and environmental contexts under a One Health framework. A better understanding of its ecological distribution and genetic plasticity is essential to inform coordinated surveillance strategies and mitigate the public health risks associated with the continued spread of carbapenem resistance.

## 1. Introduction

The *Enterobacter cloacae* Complex (ECC) is a group of Gram-negative bacteria that are widely distributed in nature and can exist in the intestinal tract of humans and animals. With the rapid advancement of whole-genome sequencing technologies over the past two decades, the taxonomic classification of ECC has been substantially refined. ECC comprises multiple genomically defined species, including the clinically relevant species *E. cloacae*, *E. asburiae*, *E. hormaechei*, *E. kobei*, *E. ludwigii*, *E. mori*, and *E. nimipressuralis*, as well as recently described species including *E. chengduensis* and *E. sichuanensis* [1]. Advances in molecular approaches, including hsp60 sequencing, multilocus sequence typing (MLST), and average nucleotide identity analysis, have further refined the population structure of ECC. These molecular tools have revealed substantial genetic heterogeneity within the complex, which is associated with variations in virulence potential, ecological fitness, and antimicrobial resistance profiles. In hospital-based studies, ECC accounts for approximately 65–75% of infections of *Enterobacter* spp. [2]. Clinically, ECC has emerged as one of the most important opportunistic pathogens responsible for healthcare-associated infections. It is implicated in a wide range of clinical syndromes, including bloodstream infections, pneumonia, urinary tract infections, and skin infections [3]. In addition, ECC has been increasingly reported in device-associated infections and intensive care unit settings, where immunocompromised patients are particularly vulnerable [4]. Its intrinsic resistance to several β-lactam antibiotics, mediated partly by chromosomal AmpC β-lactamases, significantly limits therapeutic options and facilitates the selection of multidrug-resistant strains under antimicrobial pressure [5]. The inducible expression of AmpC enzymes further complicates treatment, as exposure to certain β-lactams may promote derepression and lead to therapeutic failure. Furthermore, the combination of intrinsic resistance mechanisms and acquired resistance determinants confers substantial adaptive advantages to the adaptive capacity of ECC under intensive antibiotic selection pressure.

In recent years, carbapenem-resistant *Enterobacterales* (CREs) have emerged as a major global public health threat. CRE infections are typically associated with high levels of antimicrobial resistance, limited treatment options, prolonged hospitalization, and substantial mortality rates [6]. Reflecting their clinical importance, CREs were classified as “critical priority pathogens” in the 2024 World Health Organization (WHO) bacterial priority pathogen list [7]. The prevalence of CRE continues to increase worldwide, including in East Asia, where rising resistance rates have been reported in China and Japan [8]. This sustained expansion not only complicates clinical management but also imposes considerable economic and societal burdens on healthcare systems. Among CRE, carbapenem-resistant *Enterobacter cloacae* Complex (CRECC) has emerged as a particularly important multidrug-resistant subgroup within Enterobacterales that poses an escalating challenge in healthcare settings [9]. Recently, epidemiological data demonstrate a worrying upward trend in detection rates. For example, surveillance from a tertiary-care hospital in China showed that the detection rate of CRECC increased from 5.5% in 2011 to 18.3% in 2019 [10]. The increasing prevalence of CRECC highlights its strong adaptive capacity and the selective pressure exerted by extensive carbapenem use in clinical practice.

The major mechanism underlying carbapenem resistance in CRECC is the acquisition of carbapenemase genes such as *bla_NDM_*, *bla_KPC_*, and *bla_IMP_*. These genes are frequently carried on plasmids and other mobile genetic elements (MGEs) [11,12], facilitating horizontal gene transfer and dissemination. The mobility of these genetic elements enables rapid inter-strain and inter-species spread, accelerating regional and even transnational dissemination of resistance determinants. In addition, co-localization of carbapenemase genes with other resistance determinants on the same plasmid may promote multidrug resistance and increase the stability of resistance under antimicrobial exposure. Notably, CRECC has also been detected in food-producing animals [13], wastewater [14], and natural water bodies [15], indicating its ecological presence across multiple compartments and suggesting potential reservoirs and exposure pathways. The identification of carbapenemase-producing ECC in livestock raises concerns regarding antimicrobial use in agricultural settings and the potential for zoonotic transmission through the food chain [16]. Meanwhile, wastewater treatment plants have been recognized as critical ecological interfaces where clinical, community, and agricultural bacteria converge, creating opportunities for genetic exchange and amplification of resistance genes [17]. Therefore, addressing CRECC solely from the perspective of human medicine, while neglecting its presence and transmission dynamics in animal hosts and environmental reservoirs, would provide an incomplete understanding of its ecological cycle and dissemination pathways. A comprehensive ecological perspective is essential to elucidate the complex transmission network underlying the persistence and expansion of CRECC.

Effective prevention and control of CRECC in healthcare, agricultural, and environmental settings are essential to mitigate its continued dissemination and associated public health risks. Given the interconnected nature of antimicrobial resistance, isolated sector-specific interventions are unlikely to achieve sustainable control. A One Health framework provides an integrated approach to antimicrobial resistance surveillance and coordinated intervention across human, animal, and environmental sectors [18]. By promoting interdisciplinary collaboration, harmonized surveillance systems, and genomic data sharing, the One Health approach facilitates identification of transmission routes, assessment of ecological drivers, and implementation of targeted mitigation strategies. Moreover, integrating genomic epidemiology with environmental monitoring and antimicrobial stewardship programs may enhance early warning capacities and reduce the risk of large-scale outbreaks. Therefore, this review synthesizes current evidence on the epidemiological characteristics, resistance mechanisms, and evolutionary dynamics of CRECC in human, animal, and environmental settings. By integrating compartment-specific findings, we provide an overview of its ecological occurrence and antimicrobial resistance patterns, while highlighting areas where molecular evidence supports potential cross-compartment dissemination and where further research is needed. Particular emphasis is placed on genomic epidemiology, plasmid-mediated transmission, and evolutionary adaptation processes that shape the global dissemination of CRECC. In addition, we identify key knowledge gaps regarding transmission pathways, ecological reservoirs, and risk factors that warrant further investigation. Through a comprehensive and interdisciplinary perspective, this review aims to contribute to a deeper understanding of CRECC ecology and to inform evidence-based strategies for surveillance, prevention, and control under the One Health paradigm.

## 2. Occurrence and Ecological Distribution of CRECC

### 2.1. Epidemiological Characteristics of CRECC in Humans

CRECC has emerged as a growing threat in healthcare settings due to its high prevalence, multidrug resistance, and persistent transmission. It is associated with a wide range of infections, including urinary tract, skin and soft tissue, bloodstream infections, and pneumonia [3]. Clinically, CRECC infections are associated with substantial morbidity and mortality. For example, a retrospective study in a tertiary hospital in northeast China reported a 30-day crude mortality of 17.4% among patients with CREC infections, indicating poor clinical outcomes and high disease burden among hospitalized patients [4]. These infections are also challenging to treat clinically, as molecular epidemiology studies have highlighted both diverse resistance determinants and complex clinical characteristics among CREC isolates [19]. Among the previous clinical CRECC isolates reported in China, approximately 94.4% carried carbapenemase-encoding genes with mainly *bla_NDM-1_* (50.0%) and *bla_KPC-2_* (38.9%) [4]. Notably, the distribution of carbapenemase types varied geographically. KPC-types dominated in North America, OXA-48-types and VIM-types were mainly prevalent in Europe, while NDM-types predominated in China [20], carried in the conjugative plasmids of IncFII, IncHI2, and IncX3. Within hospitals, CRECC has been detected in healthcare workers, contaminated medical equipment, and environmental sites such as sinks and drainpipes [21], suggesting environmental persistence and potential opportunities for intra-hospital dissemination. These sites can support biofilm formation and allow CRECC to persist and reinfect vulnerable patients. Hospital outbreaks often originate from the spread of a single clonal strain (such as ST171) [22]. In addition to clonal persistence, established clinical risk factors including prolonged hospitalization, immunocompromised status, and exposure to invasive procedures are associated with increased risk of CRECC infection [4], underscoring the importance of infection control measures targeting both environmental reservoirs and high-risk patient populations.

Although community infections of CRECC have been rarely reported, emerging evidence suggests that community-gained strains share similar resistant mechanisms with those acquired in hospitals [14]. A prospective household cohort study, supported by whole-genome sequencing (WGS), reported limited transmission of carbapenemase-producing Enterobacteriaceae, including CRECC, from recently hospitalized index patients to household contacts [23]. In addition, CRECC has been reported in companion animals (see Section 2.2), indicating that animals may serve as potential reservoirs. Close human–animal interactions may provide opportunities for cross-species exposure.

### 2.2. Epidemiological Characteristics of CRECC in Animals

Companion animals are recognized as potential reservoirs of CRECC and may facilitate cross-species transmission. For example, CRECC carrying pOXA-48-like plasmids has been isolated from dogs and cats in Germany, and significantly overlapped with the human strains in sequence and plasmid types [24]. A canine-derived CRECC ST171 carrying *bla_KPC-4_* was identified in the United States and was closely related to the clinical isolates [25]. The presence of highly concordant molecular typing profiles among CRECC isolates from animals and humans (e.g., ST171, ST286, ST544, ST61) [26] suggests clonal relatedness across compartments. In addition, a 63 kb IncL plasmid harboring the *bla_OXA-48_* gene was identified in both human and animal CRECC isolates, indicating a shared resistance vehicle [27].

Livestock and poultry breeding systems represent important ecological reservoirs of CRECC. Although the use of carbapenems is strictly restricted in animal breeding, the application of other β-lactam antibiotics may contribute to the co-selection of plasmids or bacterial clones carrying carbapenemase genes [28]. CRECC carrying *bla_VIM-1_* has been detected from pig feces and cecal samples in slaughter farms [29]. Additionally, IMI-2 producing ECC were detected in animal feed in Sweden, indicating environmental persistence and potential exposure pathways within livestock systems [16]. These findings suggest environmental occurrence and persistence.

The food chain represents a potential transmission route. CRECC strains detected in meat and dairy products have shown close genetic relatedness to clinical strains [13]. In Egypt, *E. hormaechei* carrying *bla_VIM-1_* and *mcr-9* was isolated from the uncooked beef patty [30]. In Myanmar, CRECCs carrying *bla_IMI-1_* were detected in market chicken, mutton, Chinese cabbage, roselle and water spinach [31]. Aquaculture products were another reported source of ecological occurrence, such as farmed freshwater fish, Vietnamese shrimp and clams [32,33]. Whole-genome sequencing of food-derived ECC isolates has revealed large transferable plasmids (like IncHI2) carrying multiple clinically relevant resistance genes, suggesting the potential for horizontal gene dissemination through the food chain [34].

Wild animals may contribute to the environmental dissemination of CRECC. In Vienna, CRECCs were isolated from the intestinal tissues of brown rats [35]. These rodents, which often inhabit sewage systems or feed on human waste, may acquire antimicrobial-resistant bacteria and contribute to environmental dissemination. Given their mobility and environmental adaptability, rodents could play a role in bridging environmental, animal, and human reservoirs of CRECC, especially in urban ecosystems.

### 2.3. Prevalence Characteristics of CRECC in the Environment

**Figure 1:**
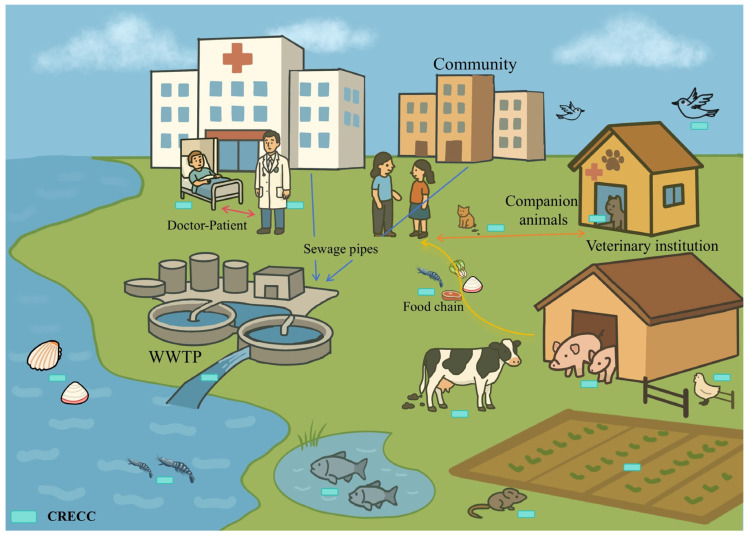
CRECC transmission pathways.

CRECC is widely distributed in environmental ecosystems, particularly in hospital wastewater, sludge, and related effluent systems (Figure 1). Wastewater systems represent one of the most significant environmental reservoirs for CRECC. Hospital wastewater typically contains high concentrations of antimicrobial residues and antibiotic-resistant bacteria, with CRECC detection rates that are significantly higher than those in community wastewater [14]. Wastewater treatment plants (WWTPs) cannot completely remove CRECC, which are subsequently discharged into surface water [17]. Notably, with wastewater irrigation or fertilizer, CRECCs carrying *bla_IMI-1_* have been isolated from plant-based foods, including coriander, basil and herbs [36]. CRECC has also been detected in natural water bodies [15]. Wild animals, such as birds and rodents, may act as carriers following contact with contaminated water sources [37]. Overall, these findings indicate environmental persistence and potential exposure pathways.

These findings suggest that CRECC has expanded beyond a single host and has become a critical ecological node in the multi-directional transmission network linking humans, animals and the environment, thereby posing a growing and significant threat to public health and environmental security.

## 3. Mechanisms of Antibiotic Resistance in CRECC

The resistance mechanisms of CRECC are complex, involving carbapenemase production, reduced outer membrane permeability, upregulation of efflux pumps, and overexpression of AmpC β-lactamases. Synergistic interactions among these mechanisms [5] enable CRECC to exhibit broad-spectrum resistance.

Carbapenem resistance in ECC can be broadly categorized into two main mechanisms [38]: (i) acquisition of carbapenemase genes and (ii) chromosomal AmpC β-lactamase overexpression or derepression combined with outer membrane porin loss. In ECC, the latter mechanism represents a well-recognized and frequently observed pathway contributing to reduced carbapenem susceptibility. Efflux pump upregulation, such as the AcrAB–TolC system, generally acts as a contributory mechanism that enhances resistance levels in combination with these primary determinants. Synergistic interactions among these mechanisms further broaden the resistance spectrum of CRECC.

### 3.1. The Emergence of Carbapenemase

The acquisition of carbapenemases represents one of the major mechanisms underlying carbapenem resistance in CRECC, particularly among carbapenemase-producing isolates (Table 1). These genes are often located on plasmids or integrons, enabling cross-species transmission. According to the Ambler molecular classification system, these enzymes can be categorized into three classes, A, B, and D, exhibiting distinct global and regional prevalence patterns [39]. Class A carbapenemases were best represented by KPC, whose genes were predominantly located in the Tn4401 transposon or its variants and were embedded within IncFII, IncN, or IncX3-type conjugative plasmids, enabling rapid dissemination among bacterial genera [40]. Among Class B β-lactamases, NDM-type enzymes were the primary type of CRECC resistance globally. *bla_NDM-1_* and *bla_NDM-5_* were frequently located in IncX3 plasmids [41] and can spread across genera via IS26 or Tn125 [42]. Moreover, *bla_VIM_* and *bla_IMP_* were typically located within Class I integrons [43]. Class D carbapenemases, represented by OXA-48, exhibited lower hydrolytic activity against carbapenems but extremely efficient transmissibility via IncL plasmids [27].

### 3.2. Non-Enzymatic Resistance Mechanisms in CRECC

**Figure 2:**
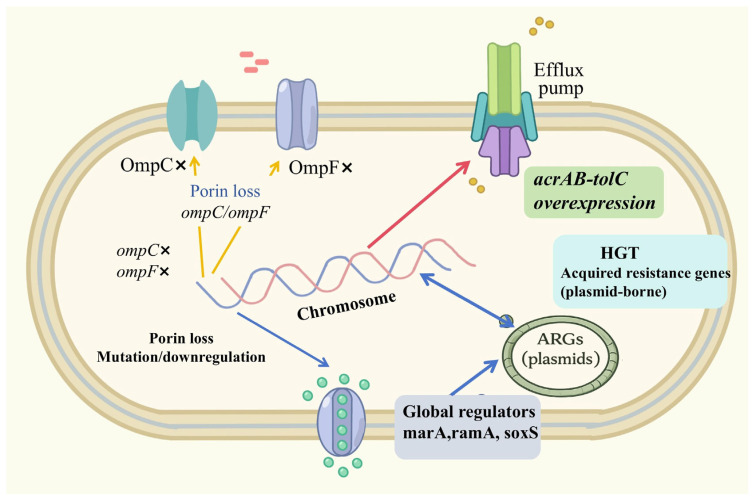
Schematic illustration of non-enzymatic resistance mechanisms in CRECC.

Non-enzymatic mechanisms also play vital roles in shaping the resistance phenotype of CRECC. Upregulation of the AcrAB-TolC efflux system increases active drug efflux and enhances antimicrobial tolerance. Similarly, mutations or deletions in outer membrane porin genes (e.g., *ompC*, *ompF*) reduce drug permeability, thus enhancing carbapenem resistance [5]. Notably, certain isolates lacking detectable carbapenemase genes still exhibited carbapenem resistance, which has been primarily attributed to chromosomal AmpC overexpression or derepression in combination with loss or alteration of outer membrane porins (e.g., *OmpC*/*OmpF*). Efflux pump overexpression may further elevate resistance levels. For instance, among 32 non-carbapenemase-producing isolates from 12 Chinese hospitals, their resistance was associated with the combined overexpression of the efflux pump and loss of OmpC/OmpF porins [60]. Furthermore, chromosomal regulatory genes such as *marA*, *ramA*, and *soxS* coordinated multiple metabolic and membrane-associated stress responses, thereby promoting adaptive resistant phenotypes [61]. Some CRECCs also acquired environmental adaptation-related genes, such as metal ion resistance genes (e.g., *sil*, *poc*, *ars*) [62] and oxidative stress defense systems (e.g., *soxR*, *katG*, *ahpC*) [63], which strengthened their environmental persistence and ecological adaptability (Figure 2).

In recent years, we found CRECC has shifted from single-enzyme analysis to a framework emphasizing multi-mechanistic interactions and population-level evolutionary dynamics. Co-carriage of multiple enzymes (such as KPC-2 and NDM-1) can confer resistance to novel β-lactamase inhibitors [60], further limiting clinical treatment options. At the genetic level, CRECC can dynamically reorganize antibiotic resistance gene modules through elements such as Tn4401, IS26, and Tn125 [40,42]. Additionally, biofilm formation by CRECC in hospital settings created concealed ecological niches and promoted long-term persistence and nosocomial transmission [64].

## 4. Evolutionary Dynamics and Interbacterial Interactions of CRECC

The evolutionary dynamics of bacteria involve a complex process, driven by molecular genetics, ecological adaptation, and microbial community interactions. Horizontal gene transfer (HGT) mediated by MGEs, including plasmids, transposons and integrons, enables recipient bacteria to acquire diverse resistance genes, including carbapenemase genes (e.g., *bla_KPC_*, *bla_NDM_*, *bla_OXA_*) and other auxiliary resistance genes (e.g., *mcr-9*) [65]. Prolonged antibiotic exposure selects for resistant variants and mobile genetic elements, which over time can alter population structure and contribute to the co-evolution of resistance and virulence traits. MGEs act as vectors that accelerate the flow of antibiotic resistance genes (ARGs) within bacterial communities, facilitating interbacterial dissemination [66].

### 4.1. MGEs Facilitate the Dissemination of ARGs

IncFII, IncHI2, IncX3, and IncL/M-type were the most frequently plasmids associated with carbapenemase gene dissemination in CRECC [67,68]. The evolution of these plasmids occurred not only via gene acquisition but also through copy number amplification and structural recombination [69]. For instance, tandem amplification of plasmid sequences can rapidly elevate carbapenemase expression levels [70]. Some plasmids can integrate into chromosomes to form stable chromosomal–plasmid hybrids, enabling bidirectional gene exchange between chromosomes and plasmids. Tn4401, Tn125, and IS26 were the most prevalent transposons within CRECC, serving as key elements for the interplasmid transposition of ARGs. Their active transpositional activity enables rapid integration of resistance genes into diverse plasmids, thereby facilitating the formation of multidrug-resistant plasmids [71].

### 4.2. Evolution of Antibiotic Resistance and Virulence in CRECC

The evolution of antibiotic resistance and virulence in CRECC exhibited characteristics of co-carriage, co-regulation, and co-adaptation. Recent genomic analyses of CRECC bloodstream isolates demonstrate that high-risk lineages such as ST171 frequently harbor both resistance determinants and virulence-associated genes [22], highlighting their potential clinical impact.

Antibiotic selection pressure is the primary force to drive the evolution of CRECC resistance, a process through sequential stages of selection, genetic drift, adaptive compensation, and dissemination [72]. This study also found that regulation of the oxidative stress response system SosRS can simultaneously upregulate the expression of efflux pump genes *acrA* and *acrB*, thereby contributing heterogeneous antibiotic resistance in ECC [73]. Furthermore, this process is significantly driven by non-antibiotic selection pressures such as pesticides, environmental pollutants, and microplastics. Heavy metals and pesticides (e.g., herbicides) accelerated the spread of bacterial resistance and virulence gene clusters through co-selection mechanisms in sewage, air, and agricultural runoff [74]. For instance, cephalosporins, quinolones, and heavy metal ions accelerated the enrichment of resistance genes via co-selection effects [75]. Microplastics, possessing potent adsorption and enrichment effects, capture bacteria and plasmids harboring resistance and virulence genes in aquatic environments, thereby promoting the co-evolution of resistance and virulence factors [76]. Co-selection pressures from antibiotic residues, pesticides, heavy metals, and other pollutants drove the persistence and dissemination of CRECCs across hosts and environments [77]. Therefore, human activities, particularly the excessive use of antibiotics and pesticides in agriculture and healthcare systems, had collectively driven the evolution of CRECC.

### 4.3. Interactions with Other Bacteria

Increasing evidence has highlighted the complex ecological and competitive interactions among bacterial species. For instance, metabolic cooperation can promote mutual growth, while mechanisms such as toxins, antibiotics, or type VI secretion systems manifested negative competitive effects [78]. These interbacterial dynamics influenced both the efficiency of resistance gene dissemination and the ecological stability of microbial communities [79]. In dynamic microbial environments, fluctuations in environmental factors (such as pH, temperature, and antibiotics) and nutrient resources caused substantial temporal variations in population abundance and community composition, disrupting the bacterial community stability and diversity [80]. Antibiotics can trigger antibiotic resistance within bacterial communities and influence their evolutionary rate and direction [81]. Certain MGEs, such as plasmids, integrating and conjugating elements (ICEs), transposons, and integrons, can carry ARGs and facilitate inter-species transfer of resistance through HGT [71]. In hospital sinks, drainage pipes, and medical device surfaces, CRECC frequently formed multi-species biofilms alongside *Pseudomonas aeruginosa*, *Klebsiella pneumoniae*, and *Acinetobacter baumannii* [82]. Such biofilm structures enhanced collective resistance and facilitated horizontal transfer of plasmids and transposons among species. Furthermore, metabolic exchange between different bacterial species may allow CRECC to survive in nutrient-poor environments, further enhancing their ecological adaptability [83].

## 5. Future Outlook and Prevention Strategies

CRECC has emerged as a significant antibiotic-resistant bacterium, possessing host adaptability, genetic plasticity, and environmental persistence. Future prevention and control must expand from clinical management to a multi-tiered governance system encompassing humans, animals and the environment, forming an integrated framework centered on the “One Health” concept. A continuous surveillance network spanning hospitals, livestock farms, and wastewater treatment systems is essential for early detection and coordinated intervention. In the clinical environment, targeted control of sinks, drainage systems and high-risk surfaces is critical. Agricultural sectors should implement antibiotic usage registration systems and safe manure treatment protocols. Environmental management should adopt advanced oxidation processes (AOPs) and ozone–UV combination technologies to reduce ARG emissions from effluent systems. Research efforts should advance the development of resistance genome databases to support risk prediction models. Future bacterial resistance control should shift from reactive responses to proactive early warning systems, and from localized containment to systemic governance. The One Health-centered resistance control network will be the key pathway to curb the spread of bacterial resistance (including CRECC).

## Figures and Tables

**Table 1:** Timeline of the discovery of carbapenemase genes in CRECC.

Year	CRECC	Types of Enzymes	Gene	Gene Location	Country	Author
1984	*E. cloacae*	A	*bla_IMI-1_*	Chromosome	California, USA	BETH A. RASMUSSEN et al. [44]
1999–2000	*E. cloacae*	B	*bla_IMP-8_*	Conjugative Plasmid	Taiwan, China	Jing-Jou Yan et al. [45]
1999–2001	*E. asburiae*	A	*bla_IMI-2_*	Plasmid	USA	Cécile Aubron et al. [46]
2000	*E. cloacae*	B	*bla_VIM-2_*	Chromosome	South Korea	Seok Hoon Jeong et al. [47]
2000–2004	*E. cloacae*	B	*bla_IMP-1_*	-	Turkey	Lalitagauri M Deshpande et al. [12]
2001–2003	*E. cloacae*	A	*bla_KPC-2_*	Conjugative Plasmid	New York, USA	Lalitagauri M Deshpande et al. [12] Simona Bratu et al. [48]
2001–2003	*E. asburiae*	A	*bla_KPC-3_*	Conjugative Plasmid	New York, USA	Simona Bratu et al. [48]
Before 2002	*E. hormaechei*	B	*bla_VIM-5_*	Nonconjugative plasmid	Turkey	Gulcin G Gacar et al. [49]
May–June 2002	*E. cloacae*	B	*bla_VIM-4_*	Conjugative Plasmid	Italy	Francesco Luzzaro et al. [50]
2002–2006	*E. cloacae*	B	*bla_VIM-3_*	Chromosome	Taiwan, China	Mei-Feng Lee et al. [51]
October 2003	*E. cloacae*	B	*bla_VIM-1_*	Chromosome	Greece	Irene Galani et al. [52]
Before September 2005	*E. cloacae*	B	*bla_IMP-4_*	Conjugative Plasmid	Australia	Björn Espedido et al. [53]
June 2007–October 2009	*E. cloacae*	B	*bla_VIM-12_*	Conjugative Plasmid	Greece	Maria Panopoulou et al. [54]
September 2009–February 2012	*E. cloacae*	B	*bla_IMP-26_*	Plasmid	Chongqing, China	Wei Dai et al. [11]
September 2009–February 2012	*E. cloacae*	B	*bla_NDM-1_*	Plasmid	Chongqing, China	Wei Dai et al. [11]
2009–2014	*E. cloacae*	B	*bla_NDM-4_*	Plasmid	United Arab Emirates	Shaimaa F Mouftah et al. [55]
Before January 2010	*E. cloacae*	D	*bla_OXA-48_*	plasmid	Istanbul	Amélie Carrër et al. [56]
2011	*E. cloacae*	B	*bla_IMP-13_*	plasmid	Spain	Yasufumi Matsumura et al. [57]
2012	*E. cloacae*	B	*bla_IMP-14_*	plasmid	Thailand	Yasufumi Matsumura et al. [57]
November 2012–August 2016	*E. cloacae complex*	B	*bla_NDM-5_*	Conjugative Plasmid	China	Chunmei Jin et al. [58]
June 2019	*E. cloacae complex*	A	*bla_IMI-16_*	Plasmid	Jiangsu, China	Jie Che et al. [59]

## Data Availability

No new data were created or analyzed in this study. Data sharing is not applicable to this article.

## Abbreviations

AOPs	Advanced oxidation processes
ARGs	Antibiotics resistance genes
CRE	Carbapenem-resistant *Enterobacterales*
CRECC	Carbapenem-resistant *Enterobacter cloacae* Complex
ECC	*Enterobacter cloacae* Complex
HGT	Horizontal gene transfer
ICEs	Integrating and conjugating elements
MGEs	Mobile genetic elements
MLST	Multilocus sequence typing
WGS	Whole-genome sequencing
WWTPs	Wastewater treatment plants
